# Seed dispersal as an ecosystem service: frugivore loss leads to decline of a socially valued plant, *Capsicum frutescens*

**DOI:** 10.1002/eap.1667

**Published:** 2018-02-27

**Authors:** Monika H. Egerer, Evan C. Fricke, Haldre S. Rogers

**Affiliations:** ^1^ Department of Environmental Studies University of California 1156 High Street, Mailstop: ENVS Santa Cruz California 95064 USA; ^2^ Department of Ecology, Evolution and Organismal Biology Iowa State University 251 Bessey Hall Ames Iowa 50011 USA

**Keywords:** bird–plant mutualisms, cultural services, gut passage, Mariana Islands, seed dispersal, traditional ecological knowledge

## Abstract

Species interactions, both mutualistic and antagonistic, are widely recognized as providing important ecosystem services. Fruit‐eating animals influence plant recruitment by increasing germination during gut passage and moving seeds away from conspecifics. However, relative to studies focused on the importance of frugivores for plant population maintenance, few studies target frugivores as ecosystem service providers, and frugivores are underappreciated as ecosystem service providers relative to other mutualists such as pollinators. Here, we use an accidental experiment to elucidate the role of seed dispersal by frugivores for maintaining a culturally and economically important plant, the donne’ sali chili (*Capsicum frutescens*) in the Mariana Islands. One of the islands (Guam) has lost nearly all of its native forest birds due to an invasive snake (*Boiga irregularis*), whereas nearby islands have relatively intact bird populations. We hypothesized that frugivore loss would influence chili recruitment and abundance, which could have economic and cultural impacts. By using video cameras, we confirmed that birds were the primary seed dispersers. We used captive bird feeding trials to obtain gut‐passed seeds to use in a seedling emergence experiment. The experiment showed that gut‐passed seeds emerged sooner and at a higher proportion than seeds from whole fruits. Consistent with our findings that birds benefit chilies, we observed lower chili abundance on Guam than on islands with birds. In a survey questionnaire of island residents, the majority of residents reported an association between the wild chili and local cultural values and traditions. In addition, we identified a thriving market for chili products, suggesting benefits of wild chilies to people in the Marianas both as consumers and producers. Our study therefore documents seed dispersal as both a cultural and a supporting ecosystem service. We provide a comprehensive case study on how seed‐dispersed plants decline in the absence of their disperser, and how to apply mixed‐methods in ecosystem service assessments. Furthermore, we suggest that scientists and resource managers may utilize fruit–frugivore mutualisms concerning socially valuable plants to gather support for frugivore and forest conservation efforts.

## Introduction

Ecosystem services are ecological functions that indirectly benefit people (Costanza et al. 1997, Daily 1997). Trophic and mutualistic interactions provide important ecosystem services by reducing herbivore pest loads (Kellermann et al. 2008, Vandermeer et al. 2010, Jedlicka et al. 2011) and increasing the production of plants that are useful to people (Kremen et al. 2007). Therefore, the decline of service providers can have ecological consequences and lead to social consequences for local human communities (Karp et al. 2013, Maas et al. 2015).

Frugivory and seed dispersal by animals is a common mutualistic interaction that benefits plants and has significant conservation implications (Tylianakis et al. 2010, Pérez‐Méndez et al. 2016). Frugivory and dispersal can affect plant recruitment by (1) increasing germination after pulp removal and scarification during gut passage (Traveset 1998), (2) enabling escape from the area of high competition and predator density near conspecifics (Janzen 1970, Connell 1971), (3) moving seeds to microsites suitable for germination (Schupp 1993, Schupp and Jordano 2010), and (4) facilitating colonization of new habitat (Nathan 2006, Padilla et al. 2012). Roughly one‐half of plant species are dispersed by animals and many of these plant species are important for a suite of regulating, provisioning, and cultural services to society (Wenny et al. 2016) ranging from provisioning of fruit, fiber, wood, and medicine (Bennett 1992) to carbon storage (Bello et al. 2015). Therefore, frugivores that sustain these plant populations are thought to be supporting ecosystem service providers (Sekercioglu et al. 2016). Despite this, few studies directly link frugivores and ecosystem services (Wenny et al. 2011, 2016). Studies that directly evaluate ecosystem services, rather than measure ecological functions, in frugivory and seed dispersal are limited to studies focused on, for example, seed dispersal with seed‐caching corvids (Hougner et al. 2006, Wenny et al. 2011, Tomback 2016) and links between seed dispersal and forest carbon storage capacity (Bello et al. 2015). This may lead to the limited recognition of frugivores in providing ecosystem services, especially in relation to other mutualists such as pollinators (Costanza et al. 1997, Chan et al. 2006, Mace et al. 2012). Reports focused on ecosystem services (e.g., the Millennium Ecosystem Assessment) largely ignore seed dispersal (Millennium Ecosystem Assessment 2005), few management criteria are informed by an understanding of dispersal services, and public perception of seed dispersal services is relatively low. In order to quantify the ecosystem service benefit provided by frugivores, seed dispersal ecology faces the challenge of mechanistically linking the dispersal process (at the beginning of the life cycle) to quantifiable benefits of plants to people (occurring years later; Kunz et al. 2011).

Understanding the links between frugivores and ecosystem services is critical because plant–frugivore mutualisms are being disrupted worldwide (Wotton and Kelly 2011, McConkey et al. 2012), which could negatively impact people through the loss of ecosystem services. Few studies have qualitatively linked frugivore declines to economically and culturally important plant species to suggest declines to human benefits, but have not quantified the loss of ecosystem services. For example, hunting of large frugivores is correlated with reduced dispersal and recruitment in trees that provide non‐timber forest products (Forget and Jansen 2007, Effiom et al. 2013). Even with robust evidence of how seed dispersal disruptions influences ecosystems (e.g., Garcia et al. 2010, Francis et al. 2012), and how seed dispersers are important for ecosystem processes that likely represent ecosystem services to society (Sekercioğlu et al. 2016), we still lack case studies that represent both of these pervasive processes.

Our goal in this study is to provide an example of seed dispersal as an ecosystem service by demonstrating that birds likely affect populations of an economically and culturally valuable plant through frugivory and dispersal. We do this in the context of seed disperser loss in the Mariana Islands in the Western Pacific. These islands offer a unique opportunity to study ecological and social impacts of seed dispersal mutualism loss because all frugivorous forest birds have been functionally eradicated from the island of Guam by the invasive brown tree snake (*Boiga irregularis*; Savidge 1987, Wiles et al. 2003). Accidentally introduced in the 1940s, the snake devoured bird eggs, chicks, and adults leading to the most comprehensive case of forest bird loss in the world. Neighboring islands of Saipan, Tinian, and Rota do not have known snake populations and still have relatively intact bird populations (Camp et al. 2009, 2012, 2014). The islands represent an accidental experiment (Hille Ris Lambers et al. 2013) that can be used to assess how frugivore absence affects fruit–frugivore interactions and human communities.

**Figure 1:**
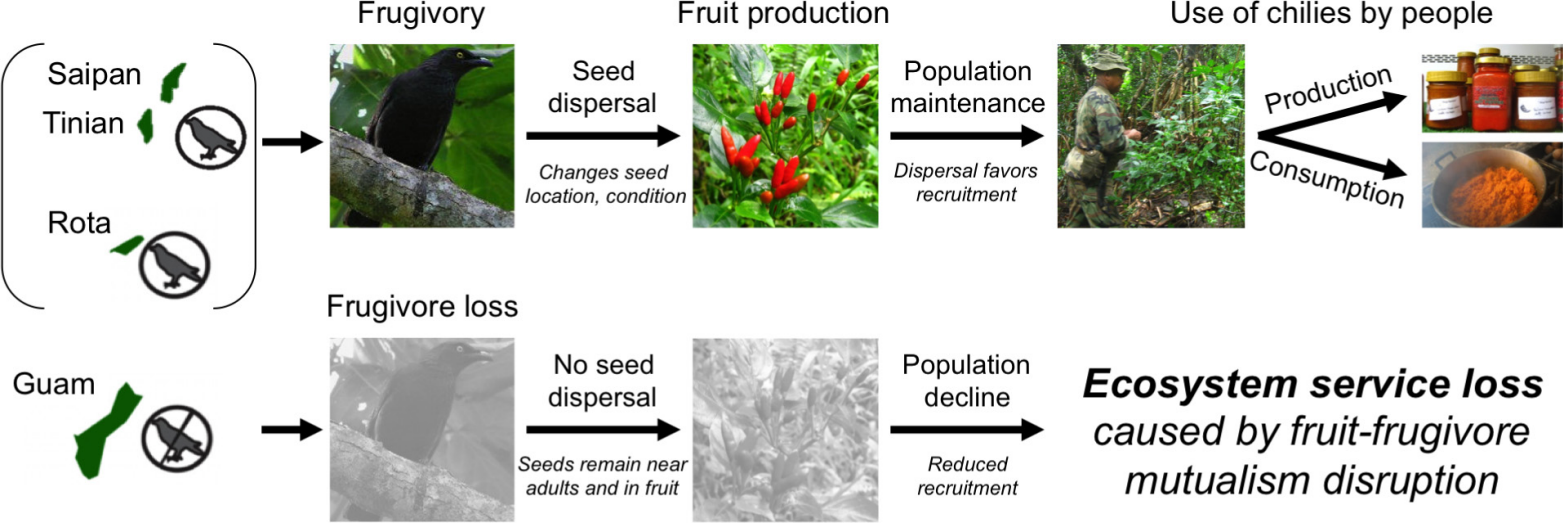
Conceptual diagram demonstrating the links between the bird–chili mutualism, its disruption, and the loss of ecosystem services. On the Mariana Islands, forest birds have been functionally extirpated from Guam but not Rota, Tinian, or Saipan. Relative to islands with seed dispersers, the loss of animal‐mediated seed dispersal may reduce germination by affecting the spatial distribution of seeds and their condition. The resulting reduction of recruitment could reduce chili populations that are economically and culturally important for people. Photos by M. Egerer.

Here we focus on an economically, culturally, and biologically significant wild plant species in the Mariana Islands: the donne’ sali chili (*Capsicum frutescens* Linnaeus). The wild chili is harvested in the forests by local people, is sold in local stores, and is a component of the local cuisine. Thus, there are monetary benefits associated with selling chili products and cultural benefits associated with consuming local and traditional foods. The chili is reported to be dispersed by the indigenous Micronesian Starling (*Aplonis opaca*), which is known locally as the sali bird, and thus the disperser is linguistically linked to the donne’ sali chili (“donne’“ means pepper in the indigenous Chamorro language). While local residents stress the importance of the Starling to chilies in the wild, there has been no investigation of this relationship and the chili may be consumed by additional bird species. In other ecosystems, frugivory and dispersal by birds has been shown to increase germination and survival of wild chilies via two mechanisms: (1) changes in seed spatial location and (2) changes to seed condition during gut passage (Tewksbury et al. 1999, Tewksbury and Nabhan 2001, Levey et al. 2006, Fricke et al. 2013, 2016). Therefore, we hypothesize that the loss of seed dispersers on Guam would negatively affect chili populations and therefore reduce ecosystem services provided by the bird–chili mutualism (Fig. 1).

We combined ecological field research with a socioeconomic assessment to investigate the importance of frugivorous birds ecologically, socially, and economically in order to understand multiple dimensions of ecosystem service provisioning, and to determine whether birds provide a service by dispersing the wild chili. In this study, we ask (1) Does fruit consumption and seed dispersal by frugivorous birds affect chili seed survival and germination? (2) Are wild chilies less common when their frugivores are absent? (3) Are chilies valued by and significant to local people? Collectively, we aimed to provide an example of a seed‐dispersal‐focused ecosystem service assessment that can inform resource management, and whose method could be applied to other systems.

## Methods

### Study system

We conducted this study on four of the Mariana Islands in Micronesia: Guam (13°27′N, 144°46′E), Saipan (15°11′N, 145°44′E), Tinian (15°N, 145°E), and Rota (14°10′N, 145°12′E; Fig. 1). All of the islands have a tropical climate with wet (July–December) and dry (January–June) seasons, and this research took place during two field sessions in both the wet (June to August 2012) and dry (December 2015 to January 2016) seasons. Forested areas of the islands are characterized by predominantly intact karst limestone forest and secondary forest dominated by tangantangan trees (*Leucaena leucocephala*; Donnegan et al. 2004). We used different islands for the different experiments, which we specify in the rest of this section.

### Wild chilies

*Capsicum frutescens* L. (Family: Solanaceae) is native to Central and South America, and is widespread throughout the Americas, Asia, Africa, and the Pacific islands (McLeod et al. 1982). The specific dispersal routes to the Mariana Islands are unknown, but the event is believed to be a part of the plant's dispersal to Japan and Southeast Asia in the 17th century with the spice trade (Yamamoto and Nawata 2005). In the Marianas, the plant is considered naturalized and non‐invasive, as it is not a common understory species on any island, does not occupy a large portion of the understory in any forest type, and does not appear to outcompete or displace other species. Rather, it is patchily distributed in lightly shaded areas under the canopy of tangantangan trees in degraded limestone forest or in gaps and along the edge of intact limestone karst forests (Fosberg 1975). Adult plants are 1–2 m tall and flower during the early spring to mid‐summer and fruits appear in late July to early December with a peak in the later months of the year (D. Fleming, *personal communication*). The elongate, pungent fruits are 1–1.5 cm in length, contain ~10 seeds, and ripen from green to red. As in other *Capsicum* species (*C. annum, C. chacoense*), birds are assumed to be the main consumers of chili fruits because birds are neurologically insensitive to the fruit's secondary metabolite capsaicin, which deters rodents and other mammals (Tewksbury et al. 1999). Donne’ sali chilies are rarely cultivated by people, reportedly because they do not grow well and are less pungent under cultivation (D. Fleming and T. Castro, *personal communication*), although other pepper varieties have been grown on island farms for over a century (Thompson 1912).

### Frugivore community

Historically, Guam had a similar frugivore community to those currently on Saipan, Tinian, and Rota (Wiles et al. 2003). Extirpated frugivores on Guam include the Bridled White‐eye (*Zosterops conspicillatus*), Mariana Fruit Dove (*Ptilinopus roseicapilla*), White‐throated Ground‐dove (*Gallicolumba xanthonura*), and Mariana Crow (*Corvus kybaryi*; Wiles et al. 2003). The Micronesian Starling (*Aplonis opaca*) and the Mariana fruit bat (*Pteropus mariannus*) are the only remaining native frugivores on Guam, however their populations are small and spatially restricted to areas protected from snakes and hunting (Rogers et al. 2012). While population trends vary between Saipan, Tinian, and Rota (e.g., the Mariana Crow is on Rota but not Saipan, and the Golden White‐eye [*Cleptornis marchei*] is on Saipan but not Rota), the frugivore communities on all three islands are more robust than those on Guam. In recent years, frugivorous bird populations have declined in abundance on Rota except for the Starling (Amar et al. 2008), maintained relatively stable on Tinian (Camp et al. 2012) and increased in abundance on Saipan (Camp et al. 2009). Unlike other regions in the world that have experienced severe bird loss (e.g., Hawaii, New Zealand), nonnative birds have not replaced the extirpated native species. We do not believe the Mariana fruit bat consumes chilies, because they tend to forage high in the canopy whereas chili plants are short (1–2 m) and because mammals tend to be sensitive to capsaicin (Tewksbury et al. 1999, Tewksbury and Nabhan 2001). As a result, our study focused solely on forest birds.

### Study design

#### Chili frugivory observations

In order to determine the frequency at which chili fruits were being consumed by frugivores, we determined frugivory rates on the island of Saipan. We marked the fruits of 10 plants with thin metal twist‐ties at each of three observation sites on Saipan chosen for their high chili abundance in the 2012 dry season. Depending on fruit availability, 10–30 red ripe fruits were marked on each plant for a total of 431 fruits spread across 30 plants. After eight days, we returned to each plant and recorded the status of previously marked fruits. Marked fruits that had an empty bract signaling complete removal or signs of consumption were classified as “eaten” fruits. Tied fruits that remained intact were classified as “not eaten.” If ties were on the ground, indicative of fruits falling off as bracts remain on the plant after fruit removal (Levey et al. 2006), they were not counted and were omitted from analysis (*n* = 9).

To record the identity of frugivores, we used video observation in July 2012 at four sites on Saipan and in December 2015 at two sites on Saipan. For each observation session, we set up a continuously running video camera for 4–7 h facing one to three heavily fruiting chili plants. We counted the number of ripe fruits on each plant before and after the session, and if fruits were missing, we reviewed the video to identify the frugivore responsible for removal. We conducted four video observation sessions for each of the six sites on Saipan, totaling of 141.5 h of observation on 55 plants.

#### Gut passage trials and shadehouse experiment

To determine whether and how gut passage by birds affects chili recruitment through increased germination, we compared the seedling emergence (timing, proportion) of seeds of different treatments in December 2015 on Saipan. We used three different seed treatments for the experiment: (1) seeds passed through the gut of captive native birds (henceforth “gut passed”), (2) seeds removed from fruit (“depulped”), and (3) seeds within whole fruits (“whole fruit”). We predicted that birds may enhance germination through gut passage by changing the condition of the seed, in which case gut‐passed seeds would have higher emergence and proportions than depulped seeds and seeds within whole fruits.

For the feeding trials, we collected ripe fruits the day before feeding trials from 5–10 fruiting adults at each of two sites on Saipan. The day of the trials, we pooled all fruits and divided them into three groups, with one randomly assigned to be fed to birds, another mechanically depulped, and the third left intact. To produce gut‐passed seeds from likely frugivores, we fed chilies to four bird species currently found on Saipan and formerly found on Guam. Outside of trials, birds were fed a species‐specific mix of seedless fruit, vegetables, and mealworms. During the trials, we offered each individual 1–40 fruits depending on the size of the species: Micronesian Starlings (four individuals) each received 10–40 fruits (total 190 fruits); Mariana Fruit Doves (three individuals) each received 5–20 fruits (total 120 fruits); White‐throated Ground Doves (three individuals) received 10 fruits (total 70 fruits); Bridled White‐eyes (one individual) received 5 fruits. We only tested a single Bridled White‐eye individual due to lack of availability of captive birds. We added chilies to the feeding bowls for each individual bird at dawn. We monitored for frugivory and seed passage hourly for up to 5 h, collecting seeds as they were passed.

Two species (Bridled White‐eye, White‐throated Ground Dove) did not consume chilies during the first trial, so we tested to see whether the lack of consumption was due to a dislike of the presentation or of chili fruit in general. We presented the recalcitrant individuals with red chilies in their bowls, as before, but added live branches with ripe fruit to their cages as well. If an individual refused to eat chilies on three separate occasions with chilies left in the cage for at least 5 h per trial, we assumed that the individual did not consume chilies.

After daily trials were finished, we planted seeds from the three treatments in an outdoor shadehouse. We first measured the average number of seeds per fruits to be 11 seeds, and assumed that number for the experiment. We planted the same number of depulped seeds and seeds within whole fruit as the number of seeds passed during each trial for each bird (Starling, 180 seeds; Fruit Dove, 240; depulped, 408; whole fruit, 216 fruits, approximately 2376 seeds). A single seed or a single whole fruit were planted individually in a plug in 72 plug trays filled with a 1:1 ratio of peat moss and perlite supplemented with fertilizer (15 g/3.8 L soil), watered daily, and monitored for seedling emergence three times a week. We recorded a seedling emergence event when a cotyledon and hypocotyl visibly emerged from the seed coat or from the degraded fruit for whole fruit treatment. We concluded the study after 11 months, because the majority of seeds had emerged, and continued emergence events were rare and not biased towards one treatment.

We conducted separate analyses to assess the impact of gut passage on the probability (proportion) and timing of seedling emergence. To analyze the effect of treatment on the probability of emergence, we used generalized linear mixed‐effects models (GLMMs) with a binomial distribution with the lme4 package (Bates et al. 2015) in R (R Development Core Team 2016). The response variable was the number of seedlings that emerged out of the number of seeds sown and the predictor variable was seed treatment (whole fruit, depulped, Fruit Dove passed, Starling passed). We include a variable describing the “fruit collection group” as a random effect to account for similarities in emergence among fruits collected on a particular day from the same set of adult plants. Because whole fruits contain a variable number of seeds that we were unable to count while keeping fruits intact, we assume that each whole fruit contained 11 seeds (i.e., number used for the depulped treatments). If more than 11 seedlings emerged from whole fruit seeds, we assumed that whole fruits contained as many seeds in the fruit as there were emerged seedlings. This occurred in nine instances (out of 216 total) and introduces a conservative bias to the analysis. We fit the full model and used the glht function in the multcomp package in R (Hothorn et al. 2008) to assess differences among all treatments in post‐hoc comparisons.

To analyze differences in the timing of seedling emergence among treatments, we used Cox proportional hazard models with mixed‐effects using the coxme package in R (Therneau 2015). The response variable was a survival variable indicating emergence status and timing, the predictor variable was seed treatment, and the “fruit collection group” was included as a random effect, as previously described. The analysis included all seeds, with right censoring of seeds where seedlings did not emerge by the end of the study period. We assessed differences in emergence timing among all seed treatments using post‐hoc comparisons with glht in multcomp (Hothorn et al. 2008).

#### Distance‐dependent and condition‐related mortality experiment

Because seed condition and escape from distance‐dependent seed predation are two primary ways dispersal benefits plants, we conducted a field experiment comparing seed predation (i.e., ratio of seed removal) between whole fruit (simulating a frugivore absent situation similar to that on Guam), gut‐passed seeds, and depulped seeds close to and distant from adult chili plants. The experiment was conducted in a tangantangan forest on Saipan, chosen because it contained a moderate density of chilies, with areas both next to and relatively far from chili plants. We selected 30 chili plants in the site for the experiment. Each of the 30 chili plants had a “near” (approximately 25 cm from stem) and “far” location (approximately 5 m following Fricke et al. 2013) from any other chili plant. We randomly assigned each plant a treatment (gut passed [10 plants], depulped [10 plants], or whole fruit [10 plants]) and added either 10 seeds (gut passed or depulped) or a single whole fruit to its near and far location. A total of 400 seeds (200 gut passed, 200 depulped) and 20 whole fruits were used in the experiment.

We placed seeds at each near and far location in piles directly on the ground using a similar approach as in previous studies of predation in *Capsicum chacoense* (Fricke et al. 2013). Seeds often fall in groups in bird droppings and the pile method thereby (1) mimics the number of seeds in whole fruits and (2) reduces the chances of confounding effects of seed number on predation. We set seeds over the course of two days because we were limited by the number of gut‐passed seeds our captive birds could produce per day. We counted the number of seeds or whole fruits remaining at each location after 7 d under two assumptions: (1) most seed predation occurs shortly after seeds land on the ground (Tewksbury et al. 1999); and (2) removal signifies predation and not secondary dispersal, as there are no seed dispersing arthropods (e.g., ants) or other likely secondary dispersers in the Marianas to our knowledge. We classified seeds that were eaten in place or removed to be dead and as alive otherwise. For whole fruits that were partially removed, we recorded the proportion of the seeds removed assuming whole fruits have 11 seeds (as in the seedling emergence experiment).

We conducted separate analyses to assess the impact of seed treatment and distance on the probability of predation by the end of the study period and the timing of seed predation. In a binomial GLMM analyzing the probability of predation by the end of the study period, the predictor variables were seed treatment (whole fruit, depulped, gut passed), distance (near or far), and a treatment by distance interaction. A variable describing the seed “pile” was used as a random effect to account for the fact that multiple seeds were placed in a single pile near or far from each adult. We fit a full model, and used glht in multcomp (Hothorn et al. 2008) to isolate benefits associated with gut passage and movement away from conspecifics with post‐hoc comparisons among treatment combinations representing the three likely outcomes for chili seeds in the field: (1) seeds are consumed and passed far from conspecifics, (2) seeds are consumed and passed near conspecifics, or (3) seeds are not consumed and remain in whole fruits near conspecifics.

To assess seed predation timing, we used a Cox proportional hazards model in *coxme* (Therneau 2015). The survival variable indicated predation status and timing, the predictor variables were seed treatment (whole fruit, depulped, gut passed), distance (near or far), and a treatment by distance interaction, and we used “pile” id as a random effect. Seeds remaining at the end of the study period were right censored. We fit a full model and used post‐hoc comparisons with glht in multcomp (Hothorn et al. 2008) to evaluate differences in the pace of removal of gut‐passed seeds near and far from conspecifics and of seeds from whole fruits.

#### Chili abundance surveys

We sought to determine whether chilies are less abundant on Guam, where dispersers are functionally absent, relative to islands with birds. Ideally, we would have identified suitable habitat and then surveyed those areas for presence/abundance of chilies. We conducted preliminary surveys on Saipan to identify habitat characteristics associated with chilies, which revealed that wild chilies are patchily distributed and capable of growing in a variety of habitat types, although most commonly in the understory of tangantangan forest. We concluded that random transects in suitable habitat would be ineffective for assessing chili populations because a large number of surveys would be required to overcome the high variability in the probability of chilies being present. Instead, we employed a suite of methods guided by existing knowledge of wild chili populations to compare chili abundance on Guam to abundance on islands with birds.

First, we used a survey method steered by local expert knowledge, including local and traditional ecological knowledge (Drescher et al. 2013), under the assumption that local users (hereby “experts”) have a valuable knowledge base of local ecology and natural resources to inform scientific research (Olsson and Folke 2001). Further, we assumed that people with a vested interest in chilies will be the best indicators of chili presence and abundance, and that the knowledge of these experts is similar between islands. Though with limitations (e.g., potential biases and lower sample sizes), local expert interviews have been endorsed as an effective method to monitor wild harvested plant species (Jones et al. 2008), especially for patchily distributed species (McGraw et al. 2003).

On each island, we interviewed local residents and organizations about historic and current locations with high chili abundances; sources of experts included the Commonwealth of the Northern Mariana Islands Division of Fish and Wildlife, Guam Department of Agriculture, University of Guam, Senior Citizen Centers, farmers’ markets, and experts suggested by other individuals in the community. We then surveyed geographic areas and sites suggested by experts where (1) people presently collect wild chilies, (2) people collected wild chilies in the past, and/or (3) chilies were suggested to be abundant (Guam, *n* = 12 sites, *n* = 10 experts; Saipan, *n* = 8, *n* = 7; Tinian, *n* = 11, *n* = 5). Experienced chili harvesters would accompany us to each site when possible. At each site, we determined the potential area of wild chili range based on suitable habitat characteristics determined from preliminary surveys including tangantangan forest, edge habitat, low canopy density, and low growing understory vegetation species (e.g., bracken ferns). Then, choosing a random starting location and direction within each area, we surveyed chili plants along a 50‐m line transect. We recorded adult chilies (woody stem intact) within 1 m on each side of the 50‐m tape to quantify the abundance of a 100‐m^2^ area. On Guam, we avoided the core area where Starlings are still present, Andersen Air Force Base, because we lacked access to the site and it did not represent a bird‐free area for the inter‐island comparison. We conducted surveys on Rota as on other islands, but the number of surveys was limited due to logistical constraints, and because local experts (*n* = 2) identified few locations (*n* = 4). As a result, we omitted Rota from analysis, but qualitatively discuss results from the few surveys on Rota.

We utilized two additional methods to assess chili abundance on Guam because we observed both a lack of wild chilies in the suggested areas and a decline in human gathering activity and therefore scarcity of local expertise on Guam. While the lack of chilies and local chili harvesters is consistent with the hypothesis that chilies are declining on Guam, it is also possible that the lack of local expertise has occurred for reasons unrelated to chili abundance. Our first supplemental method utilized collections in the University of Guam Herbarium (from the years 1975–1986) to identify locations (general vicinity, no coordinate data provided) where chilies had been collected in the past (*n* = 5 specimens). We employed the same methodology for each supplemental survey as for suggested site surveys; we visited the forested area of each specimen, delineated the expanse of suitable chili habitat (using indicators of suitable habitat characteristics described above from surveys on other islands), and then conducted a 50‐m line transect survey. If no chilies were found along the transect, we expanded the region to search for any chilies present in the patch of suitable habitat. In our second supplemental method, we searched potential areas on Guam that had habitat characteristics indicative of chili presence on other islands (habitat indicators described above). For each area of suitable habitat, we took note of the approximate distance covered, and chili presence or absence within the surveyed area. We surveyed eight additional sites on Guam, covering a total of 30,650 m^2^ of suitable chili habitat.

We used generalized linear models to compare chili abundance among islands. We included sites suggested by experts (all islands) and historical sites (Guam only) in the analysis, and omitted results from searches of suitable habitat to maintain consistent methods grounded in expert knowledge. The number of chili plants observed per site survey (i.e., 50‐m transect data) was the response, and island was the sole predictor. We fit models specified with a negative binomial error distribution to account for overdispersion using the pscl package in R (Zeileis et al. 2008). We fit the full model, and ran a post‐hoc test using the glht function in multcomp (Hothorn et al. 2008) to assess differences between islands.

#### Social value and importance

To assess the social importance of wild chilies in Mariana society, we conducted surveys among adult (>18 yr) CNMI residents (i.e., excluding tourists) in December 2015 using two methods on Saipan. A single interviewer (M. Egerer) visited 10 supermarkets, grocers, and neighborhood markets on Saipan, randomly selected from the phone book, and conducted an oral survey questionnaire with willing participants. The interviewer visited stores on weekends between 10:00 and 17:00 for 1 h at a randomly determined time under the assumption that many residents shop on the weekends. The 10‐question questionnaire consisted of yes/no and open‐ended questions and took 5–10 min. The interviewer asked whether respondents knew of the wild chili, had harvested the chili, knew of food and non‐food uses for wild chilies, if the chili was of importance in the Marianas for people (for culture, livelihoods), and if they knew what consumed chilies in the forest. We did not ask for demographic information, however, these respondents likely represented the general population of Saipan, which consists of 21.6% Chamorro (native to the Marianas), 35.8% Filipino, 7.1% Chinese, and 5.1% Carolinian as major ethnicities (U.S. Census Bureau 2010). We additionally conducted random telephone surveys using the same oral survey questionnaire format to further reach CNMI residents. For our second method, we randomly selected phone numbers from the CNMI phonebook and called households from 17:00 to 19:00 on weekdays when we assumed most residents would be home. Phone surveys did not result in high sample numbers, however, because the phonebook had not been updated after the 2015 typhoon and many numbers were disconnected. For both in‐person and telephone interviews, we conducted surveys in English, and omitted respondents where there was a significant language barrier (many residents are from the Philippines or Southeast Asia and speak little English). We combined all in‐person and telephone survey responses and calculated summary statistics (percent totals) for each answer for each respective question and summarized additional supplementary commentary that respondents provided during interviews.

To determine the economic importance of chili products on the islands to peoples’ income and livelihoods, we gathered information using (1) market surveys on Tinian, Saipan, and Guam where chili products are sold and (2) follow‐up telephone interviews with producers and other stakeholders on Tinian, Saipan, Guam, and Rota. First, we systematically visited all farmers’ markets (if present), two supermarkets, and seven small grocery/convenient stores on each island. We recorded the identity (wild or cultivated chili species) and price of chili products produced in the Marianas, including chili paste, chili sauce, and fresh chilies. Second, we contacted the producers to confirm what chili variety was used in their product and to get estimates of sales, income, and relative contribution of chili products to overall household income. We combined all market surveys across all islands and calculated the mean price for wild and cultivated products. Then, modeling all prices as a function of variety in a linear model, we used the glht function in multcomp (Hothorn et al. 2008) to determine if there was a significant difference between product varieties containing wild chilies and those containing only cultivated chilies. We used information from producers and other stakeholders (e.g., the Mariana Tourism Authority) to qualitatively assess the potential relative significance of chilies to peoples’ income and to assess potential indirect sources of income related to chili peppers.

## Results

### Frugivory observations

We observed frugivory at all three study sites on Saipan. On average, 13% of marked fruits were eaten (removed or showed signs of frugivory) after the 8‐d period. In 141.5 h of video observation, we recorded frugivory by Micronesian Starlings (five visits, 9.75 ± 3.86 fruits consumed per visit) and a Golden White‐eye (one visit, two fruits consumed).

### Impacts on seedling emergence

**Figure 2:**
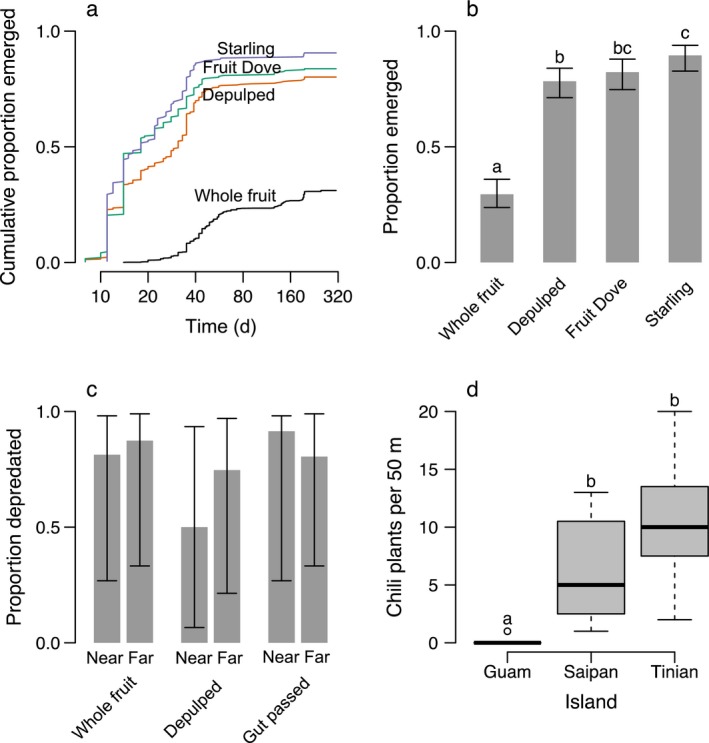
(a) The portion of chili seedlings that emerged over time and (b) the probability of emergence by the end of the study period in shadehouse experiments with seeds that were either planted within whole fruits, manually depulped, or gut passed by Starlings or Fruit Doves. In panel b, bar heights indicate model estimates under each treatment, error bars indicate Wald confidence intervals, and different lowercase letters indicate statistically significant differences assessed using post‐hoc tests (*P* ≤ 0.05). (c) Portion of chili seeds removed at the end of the short‐term seed predation study. Seeds were either gut passed by birds, not gut passed, or in whole fruit, and placed near or far from conspecific adults. (d) Wild chili counts for 50‐m survey transects on each island. Midline of box plots show the median number of plants observed per transect, box edges show lower and upper quartiles, whiskers show the maximum and minimum number of plants, circles show outlier observations. Letters within panels indicate statistically significant differences assessed using post‐hoc tests (*P* ≤ 0.05).

The Bridled White‐eye and White‐throated Ground Doves were offered fruit at least three times during our feeding trials with captive birds, but did not consume fruit, therefore gut‐passage studies were completed only with Starlings and Fruit Doves. Treatment (gut passed, depulped, and whole fruit) was a significant predictor of seedling emergence (Table 1). The proportion of seeds that emerged as seedlings was greater for seeds passed by Starlings (model mean 0.91, Wald confidence interval 0.83–0.94; observed proportion 0.91) and Fruit Doves (model mean 0.84, Wald confidence interval 0.75–0.88; observed proportion 0.84) than for seeds within whole fruits (model mean 0.30, Wald confidence interval 0.24–0.36; observed proportion 0.31; Table 1). Seedling emergence for Starling‐passed seeds was significantly greater than for Dove‐passed seeds, and manually depulped seeds had significantly lower emergence (model mean 0.80, Wald confidence interval 0.71–0.84; observed proportion 0.80) than Starling‐passed seeds and similar emergence as Fruit Dove‐passed seeds (Table 1; Fig. 2b). Emergence was greater for seeds passed by birds in comparison to depulped and whole‐fruit treatments and whole fruit had a significantly lower emergence than did all other treatments (Table 1; Fig. 2a).

**Table 1:** Analysis of chili germination over time (Cox proportional hazard models with mixed effects) and the portion of seeds germinated at the end of the study period (generalized linear mixed effects models with a binomial error distribution)

Parameter	Coefficient	SE	*z*	*P*
Generalized linear mixed effects model				
Intercept (depulped)^a^	1.3	0.2	6.7	<0.001
Whole fruit^b^	−2.2	0.1	−16	<0.001
Fruit Dove^a,c^	0.25	0.22	1.2	0.25
Starling^c^	0.86	0.28	3.0	0.003
Cox regression				
Intercept (depulped)^a^				
Whole fruit^b^	−1.7	0.07	−25	<0.001
Fruit Dove^a^	0.21	0.09	2.2	0.02
Starling^c^	0.51	0.10	5.4	<0.001

*Notes:* Parameters indicate the treatments applied within the shadehouse experiments, with manually depulped seeds as the reference level. Significant differences (*P* ≤ 0.05) among treatments assessed through post‐hoc comparisons are indicated by different superscripted letters.

### Seed predation

Chili seed predation was high overall (Fig. 2c). However, we found no differences in the proportion of seeds depredated in the three focal treatment combination comparisons (Table 2, Fig. 2c). Seed predation in whole fruit near conspecific adults (representing lack of dispersal) did not differ from predation of gut‐passed seeds near conspecifics (representing seeds consumed and dispersed near parent plants; *P *=* *0.85) or of gut‐passed seeds far from conspecifics (representing bird dispersal away from parent plants; *P *=* *0.98). Similarly, there were no differences in the proportion depredated of gut‐passed seeds near and far from conspecifics (*P *=* *0.82). The timing of seed predation also did not differ among these treatment combinations (Table 2; all *P *>* *0.2).

**Table 2:** In distance‐dependent seed predation experiments, the portion of chili seeds removed at the end of the study period (generalized linear mixed effects models with a binomial error distribution) and removal over time (Cox proportional hazard models with mixed effects)

Parameter	Coefficient	SE	*z*	*P*
Generalized linear mixed effects model				
Intercept (Far, Gut)	1.5	1.3	1.2	0.24
Depulped	0.5	1.8	0.26	0.80
Whole fruit	−1.5	1.8	−0.79	0.43
Near	−0.4	1.7	−0.22	0.82
Depulped × Near	0.8	2.6	0.31	0.75
Whole × Near	1.8	2.6	0.70	0.48
Cox regression				
Intercept (gut passed)				
Depulped	−0.01	0.70	−0.02	0.98
Whole fruit	−1.06	0.69	−1.28	0.12
Near	−0.22	0.68	−0.33	0.74
Depulped × Near	0.51	0.98	0.52	0.61
Whole × Near	0.51	0.99	0.52	0.60

### Chili abundance

Of sites suggested by local experts, wild chilies were significantly more abundant and found in higher density on Saipan and Tinian than on Guam (Fig. 2d; Tukey HSD). Guam had significantly fewer chilies than Saipan (*P *<* *0.001) and Tinian (*P *<* *0.001), but Saipan and Tinian did not significantly differ from one another (*P *=* *0.129). We found chilies in only one out of the four sites visited on Guam with a local expert. The site was at the southern end of the island, where a few starlings can be seen during the day; they likely roost at night on a small, snake‐free island offshore. Further, 7 out of 10 experts on Guam suggested searching for chilies on Andersen Air Force Base, where the last remaining Starling population exists. One expert specified that wild chilies are still present in certain areas on base and they had collected chilies there 1 yr prior (T. Nelson, *personal communication*.).

In revisiting the five sites on Guam where specimens were collected between 1975 and 1986 for the University of Guam's Herbarium, we did not find any present‐day chili populations, though sites retained historical habitat descriptions. Similarly, we did not find any chili populations by searching adequate habitat.

### Social perceptions and values

We interviewed 147 island residents on Saipan using store interviews (*n* = 129) and phone surveys (*n* = 18). We found that 71.4% of respondents knew of the wild chili (or “donne’ sali”), 42.9% had picked the chili themselves in the wild and 61.9% had eaten the wild chili or a food with the chili. Further, 65.3% of respondents reported that the wild chili has significance in Marianas culture and 53.7% reported that the wild chili is important for some islanders’ income or livelihood. People reported preparing and eating foods with wild chilies (*n* = 30; e.g., using the plant's leaves for soups and traditional local dishes), and using the plant for medicine (*n* = 12). When asked what eats the chili fruits in the forest, 44.2% of respondents identified birds as chili consumers. Of these respondents, 8.2% specified the Starling and 6.8% mentioned seed dispersal in their answer.

Chili products were found on all of the islands where we conducted the economic survey (Saipan, Tinian, and Guam). In a comprehensive attempt to identify all chili product producers, we identified a total of 14 producers that sell and distribute processed chili pastes, sauces, and pickled products. However, only four (three on Tinian, one on Guam) of the 14 used wild chilies as the main ingredient in their recipes (28.6% of producers). The remainder use domesticated chili species, including *Capsicum frutescens gossom* and *Capsicum chinense* that they cultivate in home gardens or farms. Yet, several of the producers that we interviewed said that wild chilies are more valuable than domesticated chilies or other chili varieties, but are difficult to grow or use for mass production. Wild chili seeds collected from the forest and cultivated were reported to grow with sickly yellow leaves (likely from fungal pathogens) and attract ants and small herbivores (C. Castro, *personal communication*; T. Castro, *personal communication*). Furthermore, even if cultivated plants survived to a mature fruiting adult, many reported that these chilies were not as spicy as those in the wild and thus not as valuable for products (R. Camacho, *personal communication*).

Across all islands, two of the three farmers’ markets, 40% of supermarkets (*n* = 2), and 65% of small convenience stores (*n* = 11) we visited sold wild chili products. On Tinian, wild chili products were found in all stores (*n* = 4), and all products were locally made on island. The supermarket on Tinian also advertised buying wild donne sali’ chilies for US$8–10 per pound (1 pound = 0.45 kg) for a mix of red and green and US$10–12per pound for exclusively red chilies. Prices varied across islands and among retailers and products, but in general wild‐chili‐based products were more expensive than cultivated‐chili‐based products: we found a ~50% higher price for wild‐chili‐based products (US$6.35) than for cultivated‐chili‐based products (US$4.25) when comparing a standard five‐ounce jar (1 ounce = 0.028 kg) of chili paste. Based on interviews with 10 producers across the islands, income from chili product sales was reported to range from US$500 to US$2,000 per month without accounting for labor. Further, Tinian is host to the annual Hot Pepper Festival, which brings thousands of guests and thousands of dollars to the Island (Mariana Tourism Authority, *personal communication*); and Guam also hosts the Mangilao Donne (Pepper) Festival, an annual celebration of local food and culture that provides the opportunity for vendors to sell homemade chili products.

## Discussion

Fruit–frugivore seed dispersal interactions are ecologically important mutualisms in forest ecosystems, yet we have few good examples for how seed dispersal benefits plant recruitment and in turn benefits human communities. Our study suggests that avian frugivores provide an ecosystem service through frugivory of culturally and economically important wild chilies, and that the loss of birds on Guam has led to a decline in the abundance and distribution of this plant (Fig. 1). Bird dispersal provides a benefit to chili plants through increased seedling emergence of gut‐passed seeds in comparison to depulped seeds and whole fruits. We did not find a benefit associated with seeds escaping mortality near their parent tree, and did not test other dispersal‐related benefits provided by birds, but we anticipate birds would also enable chilies to colonize new areas. The loss of birds from Guam may be responsible for the decreased wild chili abundance on Guam due in part to lack of avian gut passage; this is supported by the anecdotal information that the best remaining chili populations can be found where starlings remain. We found that the wild chili has a significant social and economic value to local residents; the chili is important in the local cuisine and food traditions, and contributes economically through sales of chili products and spending associated with chili festivals. Further, we found that the relationship between birds and chilies is present not only in the linguistic history (donne’ sali, linking it to the sali bird or Micronesian Starling), but also in narratives concerning the ecology and social perceptions of wild chilies among Islanders, leading to a story of mutualistic synergisms among birds, chilies, and people.

### Birds and chilies

Chili frugivory in our system is a rare and sporadic event as in other *Capsicum* species (Levey et al. 2006), yet the multiple frugivory events by the Starling (feeding trials, in the wild), Fruit Dove (feeding trials), and the Golden White‐eye (in the wild) confirm that birds eat chilies in this system. Chili fruits can stay ripe on plants for many weeks, implying that the overall probability of fruit removal is higher than the frugivory (~13% of fruits removed per week) that our study captured. We found that plants benefit from frugivory via increased seedling emergence after gut passage; the probability of emergence for gut‐passed seeds was approximately three times higher than the germination probability of seeds from whole fruits. Seedling emergence was also substantially faster for gut‐passed seeds than for depulped seeds and seeds from whole fruits, meaning that undispersed seeds are exposed to much longer periods of seed predation and infection prior to germination. Parental escape is considered a major advantage of dispersal for some species (Howe and Smallwood 1982), and gut passage increases seed survival and germination by removing predator attractants and pathogens for another chili species, *Capsicum chacoense* (Fricke et al. 2013, 2016). Our field experiments did not identify differences in seed predation among our seed condition and location treatments, but did show high frequencies of post‐dispersal predation in this species. These predation rates may extend over longer periods, as they do in *C. chacoense* (Fricke et al. 2013, 2016). Gut‐passed seeds germinated more quickly than did seeds in whole fruits, with half of the final germination occurring within roughly two weeks for gut‐passed seeds and two months for seeds in whole fruits; typical whole fruits are thus subjected to periods of post‐dispersal predation roughly four times longer than gut‐passed seeds. Therefore, the timing of seedling emergence following gut passage likely confers an advantage in the field in addition to the higher probability of seedling emergence identified by our shadehouse experiments. We did not test the benefit of dispersal for colonizing new habitat (Nathan and Muller‐Landau 2000), although this is likely to be another important benefit for a patchily distributed species (Eriksson and Ehrlén 1992).

The decreased abundance of chilies in the absence of forest birds on Guam is evidence that birds are providing an ecosystem service through their mutualism with chilies. On Guam, local experts and historical accounts both corroborate the finding that wild chilies have declined or are absent in habitats where they were once common. Many of the local experts we interviewed linked wild chili population decline to the extirpation of birds on Guam, and an artifact of the invasive brown tree snake. Despite similar habitat characteristics that were hospitable to wild chilies on other islands with birds (e.g., tangantangan and secondary forest), we found fewer chilies on Guam than on Saipan and Tinian. Few to no chilies were found at sites that local experts suggested, and our additional survey methods of indicator chili habitat also revealed no chilies. The sole remaining populations appear to be in locations where Starlings still remain: Anderson Air Force Base, where snakes are heavily controlled, and Merizo, where Starlings forage from their home base on snake‐free Cocos Island. It is possible that differing predator or pathogen populations on Guam limit chili populations on Guam, however, similar survival of seeds and seedlings of other plant species between Guam and other islands (Rogers et al. 2017; *unpublished data*) and the presence of chilies in snake‐controlled areas on Guam suggests that seed dispersal mutualism disruption, rather than other post‐dispersal limitations, explains low chili abundance on Guam.

We found a surprisingly low number of chili plants on Rota, even though starling populations are the greatest on Rota (Amar et al. 2008, Camp et al. 2012) and local experts did not speak of declines. The low abundance may be an underestimate caused by the small sample size, as we were limited to four sites on the island. However, few to no chilies were observed at these sites suggested by the two local experts. In addition, the lack of a local market indicates there may be a low supply of chilies. We propose two reasons for this. First, chilies may be habitat limited on Rota, since the island contains less tangantangan secondary forest (1.5% land cover) than on Tinian (34%) and Saipan (18%; Donnegan et al. 2011), in which chilies are typically found. Alternatively, chilies may be limited by the Cuban slug (*Veronicella cubensis*), an invasive pest introduced to Rota from the Caribbean Islands 15 years ago. The impacts of the slug on Rota are not well known, though *V. cubensis* exists on Rota in high numbers and feeds on *Capsicum* leaves and branches causing plant mortality (Robinson and Hollingsworth 2005). We observed *V. cubensis* feeding on wild chili plants and found plants without leaves, fruits, and weak branches at suggested survey sites on the Island. The infestation of the slug pest on Rota may explain low wild chili numbers and distribution in comparison to other islands with birds.

### People and chilies

Our results show that (1) wild chilies have a substantial social and economic value, (2) birds provide an ecosystem service via frugivory and dispersal, and thus (3) chili population decline caused by bird loss could have negative economic and social effects. First, we found that the wild chili has a significant place in the dietary patterns and traditions of many residents of the Marianas, and has a higher market value in finished products in comparison to cultivated products. In our conversations and interviews with chili experts and island residents, the wild chili can spark a passionate discussion on calibers of hotness, local food dishes rooted in diverse flavors, and a unique environmental and ethnobotanical history of human use. Our findings are based on a subset of the island population and may represent those of certain cultural traditions, but nonetheless the results provide support for the value of the chili in the social‐economic context of the Marianas. On Tinian, for example, the wild chili economy provides an alternative livelihood where there is little industry and job security. Further, Tinian hosts an annual Hot Pepper Festival that brings thousands of dollars to the Island (Mariana Tourism Authority, *personal communication*). Thus in the Mariana Islands, the high demand and supply for hot chili products in the market and the local culture and events surrounding chilies reflects a strong economic and social value associated with the wild chili.

Last, we recognize traditional ecological knowledge methodological approaches as an insightful means to glean and incorporate local knowledge and expertise in scientific research. In our study, local ecological knowledge can be ahead of scientific knowledge: local experts and residents pointed to the importance of the birds to chili abundance and distribution in the wild before our study. Thus experts in ecological knowledge come in a diversity of forms and clearly exist on a wide spectrum (Drescher et al. 2013).

## Conclusion

This study provides an example of how frugivorous birds can be ecosystem service providers through seed dispersal mutualisms and how disruptions to these mutualisms can have consequences for ecosystems and society. This work joins a growing list of ecosystem services provided by birds that include carbon storage (Bello et al. 2015), tourism (Sekercioglu 2002), and pest control in agriculture (Van Bael et al. 2008). Furthermore, this study adds another example of how seed dispersal mutualism disruptions due to habitat loss (e.g., Garcia et al. 2010, García et al. 2012), defaunation (e.g., Bello et al. 2015) or habitat degradation (e.g., Francis et al. 2012) impacts ecological function. Here we use a socially valued wild chili in the context of frugivore defaunation to demonstrate these pervasive processes in tandem: the loss of fruit–frugivore mutualisms can affect plant populations to thereby affect supporting services, and can affect local cultural traditions and livelihoods to thereby affect provisioning and cultural services. Thus, the cultural component attached to the naturalized wild chili could garner public support and recognition for forest conservation by land managers in the Marianas. As the tropics face rapid biodiversity loss in face of development, culturally important wild plants may offer land managers a tool to slow this process. Conservation and land management grounded in existing synergies between local human communities and forest landscapes can prevent biodiversity loss by utilizing the cultural dimensions of species interactions (Van Oudenhoven et al. 2011). To conclude, forest conservation is one way to fill a missing link in the bird–chili–people mutualism framework: how people may provide a benefit to both birds and chilies through forest conservation.

## Data Availability

Data associated with this paper have been deposited in a Zenodo digital repository (from GitHub) http://doi.org/10.5281/zenodo.1079881.
